# Ubiquitous skill opens opportunities for talent and expertise development

**DOI:** 10.3389/fspor.2023.1181752

**Published:** 2023-05-03

**Authors:** Duarte Araújo, João Roquette, Keith Davids

**Affiliations:** ^1^ CIPER, Faculdade de Motricidade Humana, Universidade de Lisboa, Cruz Quebrada, Portugal; ^2^ Sport & Human Performance Group, Sheffield Hallam University, Sheffield, United Kingdom

**Keywords:** sport, social, ecological dynamics, learning, expert performance

## Abstract

In this article we aim to define and present the complementary nature of talent, skill and expertise. Human daily life is replete with expressions of skillful behaviours while interacting with the world, which in specific socio-culturally defined domains, such as sport and work, demand a specialization of such ubiquitous skill. Certain manifestations of ubiquitous skill are identified by experts from the specialized domain of sport with the label of “talent”. In this paper we propose that “talent” is thus socially defined, considered identifiable at an early age and forms the basis for selection and entry at the starting point in domains like sport. Once an individual, defined as “talented” enters the “pathway” for participating in the sport domain, there begins an intense socialization process where training, evaluation, institutionalization and framing takes place for continued development of such talent. This is the formalised process of working on ubiquitous skills refining and changing them into specialized skills in sport. An ecological dynamics rationale is used to explain that this specialization approach is developed through a process of expert skill learning, which entails the stages of exploration and education of intention stabilization and perceptual attunement, and exploitation and calibration. Skill learning aims to develop potentiality and its expression in actuality, i.e., how learning is expressed in contextualized expert performance.

## 1. Introduction

A pressing issue for scientific and sport communities is to understand how skill learning supports and shapes the continued development of talent and expertise in a specific domain (1, 2). Although, as Baker and colleagues (3) have argued, concepts like talent, skill, expertise and performance tend to be used in an overlapping way in the sport sciences domain. What these concepts mean is often far from clear and a key source of confusion is the lack of socio-cultural framing of these concepts.

Collins and Evans (4, 5) have proposed the idea of expertise as a socialization process, distinguishing between ubiquitous and specialised skill. Here, we explore the insight that contextualized ubiquitous skills form the foundation of social life, facilitating the socialization process in specific specialist and expert groups. Ubiquitous skill can be expressed by every individual, consequently opening possibilities and providing a necessary basis for participation in more specialized or expertise programmes. In this paper we discuss this position, seeking to explore how skill learning contributes to talent and expertise development. We start by clarifying how skill is *ubiquitous* to human activities, examining how ideas on talent and expertise need to be framed into specialised socio-cultural domains. The key idea that we discuss is that skill in not an “entity” that can be “acquired” and “possessed” by an individual but rather is contextually defined, providing an “adaptive, functional relationship between an organism and its environment” (6, pp.18).

## 2. Expertise is socialization into a specific domain

Socialization is foundational for expertise (4) since expert performance is realized in specific social settings. Performing skillfully is not something an individual or a team possesses to begin with, but is a relational tendency that emerges to become more stable with practice and experience and is expressed and adapted in a given social setting. This socialization process has, according to Carr (7), four main properties: (i) Training, undertaken socialization practices through which novices are initiated with that culture; (ii) Evaluation, captured as methods to distinguish among expertise levels in social settings where those practices are performed; (iii) Institutionalization, indicating how expert knowledge (specializations, and differentiations) is formalised, stabilized and certified in institutions and everyday practices; and (iv), Framing or naturalization, which identifies manifestations of expert performance as bodies of knowledge, highlighting cultural and historical assumptions embedded within dominant forms of expertise, framed as evidence of performing skillfully.

This extensive process of socialization in a specific cultural domain that characterizes expertise implies participation, i.e., experience and engagement in relevant social practices (5). Social embedding when developing skill needs to be specified in an expert community to be considered as specialization. Consequently, ubiquitous skills, such as bipedal walking, are not framed into an expert community, contrasted with race walking which depends on voluntary participatory immersion in a *form of life* (8) in an Athletics organization. A manifestation of skill in race walking is a specialization of a skill, which contrasts with the ubiquitous skill involved in daily walking to navigate through everyday environments. Importantly, specialized skills are socio-culturally defined by an expertise community (e.g., national and international Athletics federations).

Expertise is expressed in, and sustained by activities of a social group. Therefore, the distinction between ubiquitous skill and expert skill could be construed as a sociological distinction, not an epistemological nor biological distinction. Such a distinction led Collins and Evans (5) to call those specialized skills practiced by a small community *esoteric*, such as skills for performing abstract algebra procedures in mathematics or for performing a triple jump in athletics, contrasting with ubiquitous skills such as counting when shopping or jumps in the backyard.

Developing skills needed to become an expert means becoming a full and active member of a social group and learning to act in ways that go beyond what novices can achieve. Collins and Evans (5) rightly argued that expertise is both context-sensitive and dependent on tacit knowledge. Only context-specific socialization can enable an individual to share and use the collective knowledge of the group and so develop (tacit) skills needed for future circumstances.

## 3. Talent as the starting point for entering into an expert domain

To overcome the lack of clarity in conceptualising “talent”, Baker and colleagues (3) present it as the starting point for the processes of learning and development that may lead to expertise. Clearly, in many instances talent is presented as a relationship to be developed (9, 10) between an individual and a specific domain. Therefore, an entry point into a socially specialized domain, implies identification and selection processes sustained by what those in a community understand as a possibility to excel, or the prerequisites and precursors of the specialised expression of skilled behaviours that characterises members of an expert group (11). Applied scientists and sport practitioners have accurately highlighted research indicating that the variables that correlate with performance at young ages are not necessarily the same variables that explain expert performance later (3), an important contribution to clarify what the notion of *talent* entails. Intriguingly, Baker et al. maintain the pervasive idea that talent can be predicted before any learning or development occurs, i.e., the idea that talent is innate. We have questioned elsewhere (9) the idea that measuring an alleged innate or foetal property at birth, or just after, is relevant for predicting sport performance potential in later life. Such properties, measured at early points in time, could be categorized as innate, but will certainly change over time shaped by the nonlinear nature of interactions with varying constraints of genes, epigenetics, experiences, surroundings and chance. These questions signal that observed skill or performance should always be understood and defined at the level of the performer-environment system (12).

In sum, talent is the starting point to entry into the expert domain of sport. This starting point emerges when performers express particularities of ubiquitous skill in occasions where expert members of a sport community can identify those particularities in sport related tasks, and consequently these performers are facilitated to enter into “pathways” of an organised domain of sport specific practices for training, evaluation, institutionalization and framing. From this time on, ubiquitous skills may be prepared to be socialised (trained, practised, integrated) in a sport domain and thus to be developed into expert skills in sport.

Clarifying what particularities of ubiquitous skill look like, implies the understanding that skills are always expressed in actions, they are part of an activity (1). Realizing an activity involves the whole organism in a dynamic transaction in the environment to achieve a task goal. Actions, thus, have to be intentional and future-oriented. Actions (i.e., goal-directed changes of body movements and postures) are guided by prospective information available in the task context (13). The perception of prospective information informs how upcoming changes in movement kinetics and kinematics can be counteracted before they perturb the dynamic flow of action. By moving, the performer learns about properties that change and properties that remain invariant, about how to coordinate with events and objects of the environment, and about information that makes it possible to guide action prospectively. In short, entangled in a complex system, actions develop through acting (13). Skill learning is the process of *sophisticating* (refining) how one acts and engages with the interconnected world, in ubiquitous or in expert domains, to achieve a task goal.

## 4. Skill is ecological (embodied and embedded)

Skills may be best defined as embedded or ecological instead of disembodied or mentally represented (14). They are not internalised and possessed by the performer, but they reciprocally characterise an emerging relationship between the whole individual and possibilities for action available in a performance context (15). Skills are part and parcel of performing in socially-defined activities (16), usually entangled with other skills, such as talking, standing, grasping, pushing or concentrating. These skills frame the experience of performing an activity in a context - such as starting, accelerating, maintaining, curving and finishing when running a lap on a track - they are not isolatable, nor they can be split into components in performance. By purposefully engaging with community activities, performing skills reveals information for affordances (i.e., action possibilities offered by the environment (17), which reveal new skills to be performed, and so on. In this way, skills are framed by *knowledge of*, rather than *knowledge about* the environment (18). From this viewpoint, the role of practice is to enhance the degree of fit between an athlete and the performance environment, instead of the enrichment of an athlete's mental representations. In this regard, the term “knowledge of” explains how to (perceive and) act, which is in contrast with verbalizable knowledge or “knowledge about” (e.g., a verbal description of performance) which may or may not correlate with a performer's contextualised manifestation of skillful performance in sport (19).

Social-cultural behaving domains (including sports) have been developed in such a way that they facilitate non-conventional behaviors, from which new skills emerge. This skill adjustment process implies a form of learning that is not based on intellectually or passively detached memorization of instructions, but by evolving bodily engagement in a task context (20). What the skill develops through experience is not represented in the mind, but it is presented to participants as more and more finely salient affordances (21). If an invitational affordance does not demand a response, or the response does not generate an intended outcome, the participant is led to further refine their perceptual attunement, which in turn, solicits more refined actions, and so on. This continuous adaptive process is not a mental evaluation of what is going on, instead it is constitutive of being corporeally engaged in the activity. In other words, acting is experienced as an ongoing process of developing skillful behaviors solicited by a task context.

This understanding of skill as a refined coupling of perception and action performed in a specific domain, challenges traditional notions of skill acquisition. These models have an explanatory preference for automatic mental processing (22) or building complex mental representations that do not support premature automatization of performance (23) to be the end goal of the skill acquisition process. Contrastingly, from an ecological dynamics perspective, a skill cannot be acquired or possessed (6). Skill learning is a non-linear process which continually refines the fit between an individual and a performance environment. To adjust performance in a sport task to the affordances of a specific task context, implies “sophisticating” *knowledge of* the environment and not the acquisition of *knowledge about* the environment (e.g., memories, fast mental processing).

## 5. Ecological dynamics of skill learning

From an ecological dynamics approach, the primary challenge facing any individual is the successful performance of goal-directed behaviors. Therefore, skill learning is more about the fine-tuning of perceiving and acting abilities than it is about the building mental representations about the world (24). Moreover, this process of fine-tuning perception, cognition and actions emerges from the refinement of the ability to detect and exploit information about affordances rather than the modification or enrichment of mental representations.

Skill performance involves perceiving an affordance, which is predicated on an individual's ability to detect information in a given environment relative to their action capabilities. As skill is developed in an (expert) cultural context, a person becomes attuned to a wider range of affordances and gains a greater sensitivity to contextual consequences of their actions (25). A stage-like model of skill learning was elaborated from the work of pioneers influencing ecological dynamics (e.g., 26–29), resulting in a non-linear three-stage model of skill learning in sport (1, 19, 30). These stages are nested together, not sequentially where one necessarily comes before the other, but can emerge at all three stages. The stages are dependent on continuous behaviours and activity, and not stored as rule-like prescriptions in the individual's mind.

### 5.1. Search: exploring possibilities

Learning which behaviors to perform, what affordances to perceive, and how to explore and discover information about those affordances is called the *education of intention* (29). Intentions shape perception–action links during skill performance. A practice environment can be designed to constrain intentions of actors, influencing which particular affordances may be perceived and when. When a performer's intentions converge on a task goal, affordances inform them how to attain the intended goal. Intention directs the attention of an actor, and stimulates exploratory behaviors that channel perception, which further constrains action, and so on in a cyclical way (31). Intention directs perception for particular affordances (1). Performers increase their exploratory actions, when it is difficult to discriminate which properties of the environment constitute information to act upon a task and which do not. Exploring what is available in a performance context is a relevant behaviour that can disclose what environmental properties are informative relative to task goal achievement (25). By exploring a task context, the intentions of a performer become constrained by the task.

### 5.2. Discover: steadying the person-environment coupling

When the performer discovers tentative “solutions”, they can maintain the person-environment link in behaviours that guide them towards goal achievement. Discovery potentiates the possibility that later the performer comes to know of task properties that change and properties that remain invariant, about how to coordinate actions with the environment, and about information that makes it possible to guide action prospectively to task goal achievement. This approach in “repetition without repetition” (26, p 234) stabilizes perception-action couplings. When the performer's intentions converge towards a task goal, the need arises to organize body movements specifically for achieving these intentions. Stabilizing body movements can be done by “freezing” corporeal degrees of freedom (32). However, with practice, corporeal degrees of freedom begin to “free up” when acting. More relevant perception-action couplings are next discovered, i.e., the conditions for *how* and *when* affordances are perceived and acted on. Perceiving and acting abilities can be fine-tuned to subtle adaptations in which specific components of a given ambient structured energy array (i.e., ecological information) are detected and exploited in perceiving an affordance. Ideally, during learning, performers will progress toward detecting information that provide more useful information about an affordance. Learning which patterns in a given structured energy array provide information about a given affordance has been called the *education of attention* or perceptual attunement (27, 31).

### 5.3. Exploit: linking with refined affordances

Changes in intention and attention often result in changes in how a performer *uses* a given ambient stimulation pattern (i.e., ecological information) to perceive a given affordance. Learning how to use the information about a given affordance to appropriately perceive a given property or perform a given behavior is called calibration (29). Exploiting perception and action supports adjustment to contextual demands. Body dimensions and characteristics are not fixed, but change across time. When body characteristics change (e.g., with practice and training), actions may become more or less challenging (33, 34). Consequently, attunement to a wider range of informational variables in a performance context, becomes important as well as greater sensitivity to contextual consequences of one's actions. Calibration involves refinement of mapping between prospective information and acting (and perceiving) (35). Continued experience leads to better calibration.

## 6. Skill learning: from possibility (talent) to actuality (expert performance)

Understanding behavior at the level of the performer-environment system means that skill is not a property located in the athlete nor in the environment, but it implies a linkage of the performers’ corporeal characteristics with affordances offered by a task context. Additional constraints are related to the personal characteristics of a specific performer who is ready to act upon an affordance. One thing is to qualify for the Olympics (a real possibility) and another is to be ready to compete on the day of the event (e.g., in excellent condition without injuries). So, the personal potentiality for acting on the affordances available in competition implies satisfying an additional layer of constraints. This potentiality is further constraining as competition starts. Actual performance is a another narrowing down of possibilities. Performers form intentions in circumstances where they are directly informed of possibilities offered to them. Out of many successful paths connecting initial conditions to a performance goal, one path emerges (actuality), although this path has already been constrained by previous skill learning experiences (potentiality) (36, 37). Skill learning is the process of developing the potentiality that links the possibility of entering in the sport domain, e.g., when a youth is identified as “talented” with potential to compete in a given sport, to that of actuality, i.e., when expert performance is expressed in elite competition.

## 7. Concluding remarks

In this paper, we explored the social foundation of sport expertise, seeking to clarify the complementary relations between talent, skill, learning and expertise. With respect to talent development, a performer intent on belonging to an expert community and identified by that community as a talent (based on their ubiquitous skills), is at the initiation point, with an *opportunity* to enter a domain of expertise, such as sport. Then, individuals expressing talent become socialized and attuned to the historical and cultural constraints of a sport to develop their expertise. This skill adaptation process is when specialized sport skill learning takes place, and the *potentiality* for expert performance is developed. However, the *actuality* of expert performance only exists when skilled behaviours are expressed in a given task context. The development of talent to expert performance is grounded on continued *sophistication* of sport skills through learning.
